# Structure–function analysis of the *Bacillus megaterium* GerUD spore germinant receptor protein

**DOI:** 10.1093/femsle/fnv210

**Published:** 2015-10-28

**Authors:** Srishti Gupta, Ke Xu Zhou, David M. D. Bailey, Graham Christie

**Affiliations:** Department of Chemical Engineering and Biotechnology, Institute of Biotechnology, University of Cambridge, Tennis Court Road, Cambridge CB2 1QT, UK

**Keywords:** *Bacillus*, spores, germination, germinant receptor, D-subunit

## Abstract

Germination of *Bacillus* spores is triggered by the interaction of germinant molecules with specialized receptor proteins localized to the spore inner membrane. Germinant receptors (GRs) are comprised typically of three interacting protein subunits, each of which is essential for receptor function. At least some GRs appear to have a fourth component, referred to as a D-subunit protein. A number of D-subunit proteins were shown previously to be capable of modulating the activity of associated GRs. Here, we investigate the topology and structure–function relationships of the *Bacillus megaterium* QM B1551 GerUD protein, which is associated with the GerU GR. The presented data demonstrate that GerUD can be subjected to relatively extensive structural modifications while retaining function. Indeed, the presence of either of the two transmembrane spanning domains is sufficient to modulate an efficient GerU-mediated germinative response. The precise function of D-subunit proteins has yet to be established, although they may act as molecular chaperones within the spore inner-membrane environment.

## Abstract (precis)

A receptor protein involved in the germination of bacterial spores can be subjected to major structural modifications while retaining function.

## Graphical abstract

**Graphical Abstract Figure.**
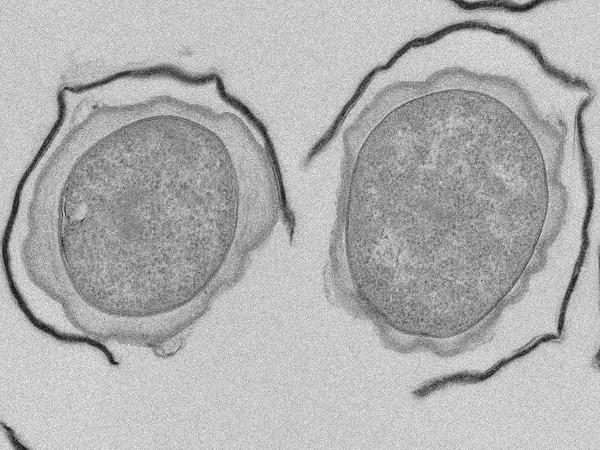
A receptor protein involved in the germination of bacterial spores can be subjected to major structural modifications while retaining function.

## INTRODUCTION

Dormant spores of *Bacillales* and most *Clostridiales* species rely upon specialized receptor proteins to sense environmental cues that are indicative of conditions that are supportive of vegetative growth and metabolism (Moir 2006; Setlow 2014). The archetypal spore germinant receptor (GR), GerA from *Bacillus subtilis*, is composed of a complex of three distinct proteins, namely the integral membrane proteins GerAA and GerAB, and the membrane-associated GerAC protein (Zuberi, Feavers and Moir 1985). All three proteins are localized to one or two clusters in the spore inner membrane, where they are thought to interact to form a complex that is responsive to the amino acids L-alanine and L-valine (Griffiths *et al*. 2011). Spores of most species are equipped with a number of orthologous GRs, which are responsive to different amino acids or other germinant molecules, including sugars and inorganic ions (Paredes-Sabja, Setlow and Sarker 2011).

Additionally, some spore GRs have a fourth component, a so-called D-subunit protein, which is encoded either within or adjacent to the main GR operon (Paredes-Sabja, Setlow and Sarker 2011; Ramirez-Peralta *et al*. 2013). Bioinformatical analyses indicate that GR D-subunit proteins are composed of two transmembrane (TM) spanning domains connected by a loop of varying length, with short N- and C-terminal regions. Given that they are expressed only in the developing forespore, it seems likely that they are integral membrane proteins that localize to the spore inner membrane (Ramirez-Peralta *et al*. 2013). Previous molecular-genetic-based analyses demonstrated that some GR D-subunit proteins could positively modulate the function of their associated GRs e.g. *B. subtilis* and *B. megaterium* GerKD, enhancing spore sensitivity to cognate germinants, while appearing not to influence the abundance of other germination apparatus proteins (Ramirez-Peralta *et al*. 2013). In contrast, deletion of *B. megaterium gerUD* was shown to exert a negative effect on the apparent affinity of the parental GerU receptor towards glucose and proline germinants. However, the molecular mechanism(s) associated with D-subunit modulation of GR function and overall spore germination rates have not been established. Hence, the objective of the current study was to attempt to gain some insight to D-subunit function by using mainly molecular genetic techniques to investigate structure–function relationships of the *B. megaterium* GerUD protein.

## MATERIALS AND METHODS

### Bacterial strains, spore preparation and germination assays

*Bacillus megaterium* strains employed in this study (Table S1, Supporting Information) were isogenic with strain GC614, a derivative of the wild-type QM B1551 strain that is null for all five functional GRs (Gupta *et al*. 2013). *Bacillus megaterium* strains were routinely cultured in LB medium at 30°C, containing antibiotics where appropriate (Table S1, Supporting Information). Spores were prepared by nutrient exhaustion in supplemented nutrient broth and purified as described previously (Gupta *et al*. 2013). Germination assays, conducted in 5 mM Tris-HCl buffer (pH 7.5) supplemented with varying concentrations (0.1–1000 mM) of glucose and or L-proline, were performed as described previously (Ramirez-Peralta *et al*. 2013). Germination kinetic analyses were performed using SigmaPlot 11.0 (Systat Software Inc.). All experiments were performed in triplicate, with at least two independent spore preparations for each strain.

### Mutant strain construction

A complementation-type approach was used throughout this work, with all mutant constructs being pHT–*gerUD*^M10stop^ gerU* derived transformants of strain GC614, where each strain carried a copy of the gerU* operon under control of its native promoter plus a modified D-subunit gene. Construction of plasmid pHT–*gerUD*^M10stop^ gerU*, which comprises truncated *gerUD*, plus ORFs for *gerUA*, *gerUC* and *gerVB*, under control of native *gerU* regulatory sequences, has been described previously (Ramirez-Peralta *et al*. 2013). DNA fragments containing variant *gerUD* ORFs were prepared by PCR, site-directed mutagenesis, or synthesized chemically (GeneArt, Life Technologies, UK), using *B. megaterium* QM B1551 genomic DNA as template where appropriate, and then cloned between the *gerUC* and *gerVB* ORFs within the *gerU* operon using the Gibson Assembly method (NEB, Hitchin, UK). The resultant plasmids were isolated from *Escherichia coli* DH5α (NEB, Hitchin, UK), verified by DNA sequencing, and then used to transform *B. megaterium* GC614 to tetracycline resistance, employing the polyethylene-glycol-mediated transformation procedure described previously (Christie and Lowe 2008). Sequence information for all oligonucleotides used in this work is available upon request.

### Heterologous expression of GerUD topology reporter proteins

Several *E. coli* expression plasmids were constructed to facilitate topology mapping of the GerUD protein. In the first, PCR was used to amplify the entire *gerUD* ORF (minus stop codon) from genomic DNA, which was inserted into plasmid pBADcLIC-GFP using a ligation independent cloning (LIC) technique (Geertsma and Poolman 2007). Expression from the resultant plasmid is designed to produce GerUD-GFP, where GFP is fused to the C-terminus of GerUD. In the second plasmid, PCR was used to prepare a DNA fragment in which the *gfp* ORF was fused between codons 26 and 43 of *gerUD* i.e. replacing the predicted loop, with the exception of two residues at either side that connects putative TM1 and TM2 domains. The resultant amplicon was similarly inserted by LIC into plasmid pBADcLIC. Overlap PCR was used to create DNA fragments designed for the expression of GerUD-alkaline phosphatase (PhoA) fusion proteins, using plasmid pHA1 (Rapp *et al*. 2004) as template DNA for the PCR amplification of the *E. coli phoA* gene. Amplicons were purified and cloned by LIC to construct pBAD-derived plasmids for the expression of GerUD-PhoA C-terminal and loop fusion proteins analogous to the GerUD-GFP constructs.

Expression experiments were conducted using *E. coli* Top10 cells for GFP fusions, and *E. coli* CC118 cells (null for *phoA*) (Rapp *et al*. 2004) for PhoA fusion experiments. In both cases, cells were cultured at 30°C and 225 rpm in 500 ml LB medium containing 50 μg carbenicillin, to which arabinose was added to 0.2% (w/v) to induce protein expression when the OD_600_ of the culture reached 0.5. For GFP experiments, samples were withdrawn for fluorescence microscopy purposes during protein expression, which was permitted to continue for 6 h. Samples of purified *E. coli* cellular membranes were subject to SDS-PAGE and western blot analyses, the latter using HRP-labelled anti-GFP antisera (Abcam, UK). Alkaline phosphatase assays were conducted using *E. coli* cells harvested 6 h after initiation of protein expression. PhoA activity was assayed using an Alkaline Phosphatase Diethanolamine Detection Kit (Sigma Aldrich) according to the manufacturer's instructions. Essentially, 20 μL of *E. coli* cells (re-suspended in 10 m mM Tris-HCl, pH 7.5; OD600 = 25) were added to 980 μL of reaction buffer and the increase in absorbance at 405 nm of the clarified suspension recorded after 15 min at 37°C.

## RESULTS AND DISCUSSION

### GerUD topology

*Bacillus megaterium* GerUD, which is encoded at plasmid-borne locus BMQ_pBM70069, is predicted to comprise 76 amino acids and have a molecular mass of 8.7 kDa. Analysis of the protein sequence with several different topology prediction and hydrophobicity profiling programs (Table S2, Supporting Information) indicate that the secondary structure of GerUD comprises a short N-terminal sequence (residues ∼1–5), and two TM domains (TM1, residues ∼6–24; TM2, residues ∼45–67), that are connected by an outward facing loop (residues 25–44). The short C-terminus of the protein (residues 68–77) is predicted to reside in the cytoplasm (i.e. spore core).

**Figure 1.**
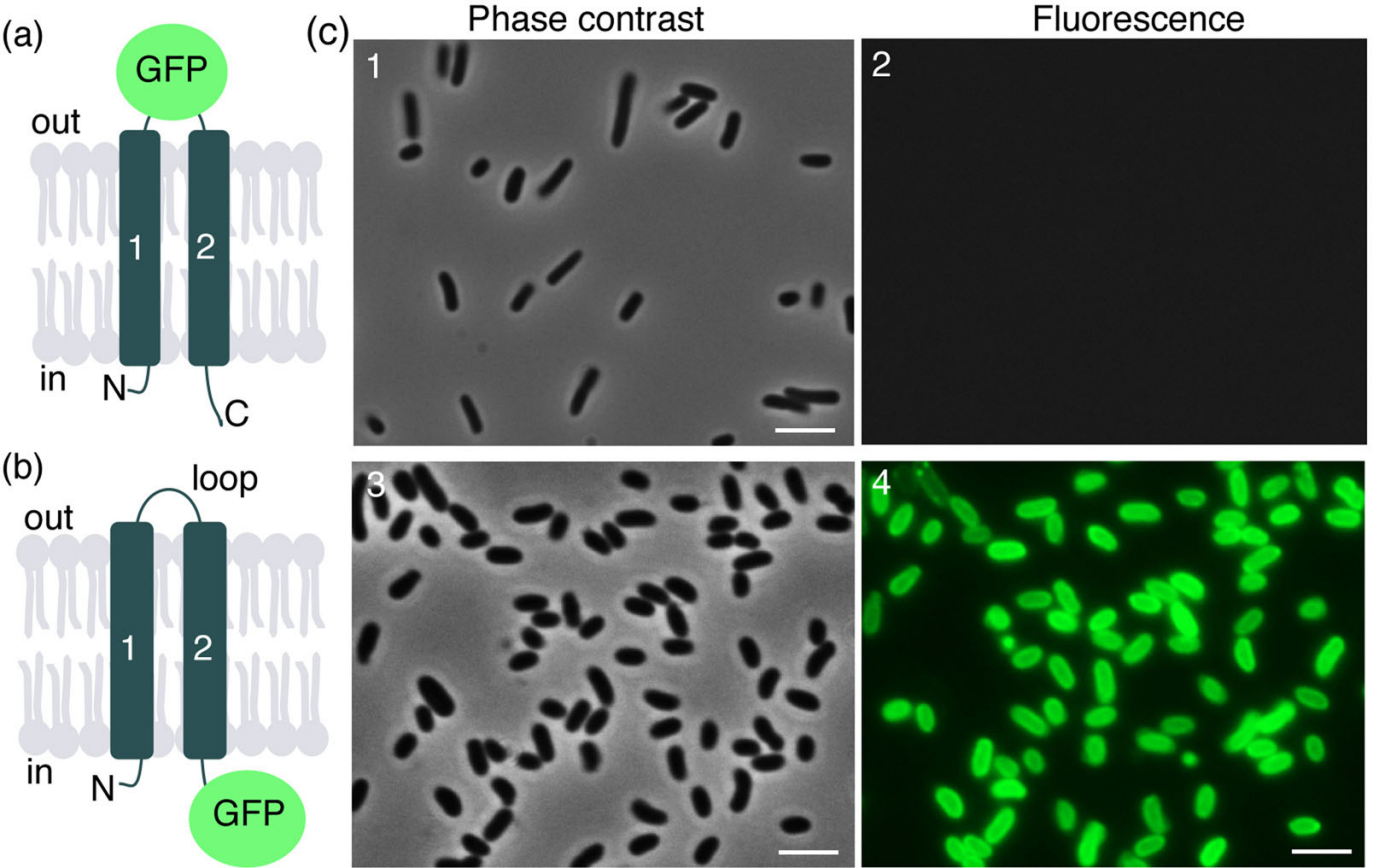
Topology mapping of *B. megaterium* GerUD. *Escherichia coli* strains designed to express GerUD with GFP located in the (**a**) TM-connecting loop, or (**b**) as a C-terminal fusion protein were examined by fluorescence microscopy after induction of protein expression. (**c**) Fluorescence associated with only the C-terminal fusion protein (image 4) is consistent with a topology in which the GerUD loop region is located in the periplasmic space and the C-terminus is cytoplasm located in *E. coli*, as shown in cartoon (b). Scale bars represent 5 μM.

In an attempt to test these topological predictions experimentally, plasmids designed for the over-expression in *E. coli* of various GerUD reporter-fusion proteins were constructed. In the first construct, the *gfp* gene was positioned in the predicted loop region that connects TM1 and TM2. This was anticipated to result in the expression of a GerU–GFP fusion protein in which the GFP moiety is located in the periplasmic space of the *E. coli* cell envelope. Since GFP does not fold efficiently in this environment (Feilmeier *et al*. 2000), then fluorescence would not be expected if the predicted topology of the fusion protein were consistent with reality. The second construct comprised a fusion of GFP at the C-terminus of the GerUD protein, which is predicted to be cytoplasmic, and therefore conducive to correct folding and fluorescence of GFP. Strong fluorescence observed in *E. coli* cells expressing the C-terminus GerUD–GFP fusion protein, and the absence of fluorescence in the loop-located GFP variant protein, is consistent with a model of GerUD in which the C-terminus is located in the cytoplasm, and the loop that connects predicted TM1 and TM2 is located on the outer face of the membrane (Fig. 1). The possibility remained that the lack of fluorescence of *E. coli* cells expressing the GerUD–GFP loop protein was simply due to an inability to express full-length protein, or that the fusion protein was unstable. However, a western blot using anti-GFP antibodies revealed that both fusion proteins were expressed at similar levels of abundance and at the expected molecular weight in *E. coli* (Fig. S1, Supporting Information), indicating that the lack of fluorescence in the GerUD–GFP loop-fusion protein is due to misfolding of GFP. Analysis of lysed *E. coli* cells indicated that both fusion proteins localized predominantly to the membrane fraction (data not shown).

Further evidence for a periplasmic location in *E. coli* for the loop segment of the GerUD protein was conferred by analysis of strains designed to express GerUD-PhoA fusion proteins. PhoA is active only in the periplasm since disulphide bonds crucial to its enzymatically active fold cannot form in the cytoplasm (Rapp *et al*. 2004). Hence, detection of PhoA activity in *E. coli* cells expressing GerUD where PhoA is fused between the predicted TM domains, in contrast to cells expressing the GerUD-PhoA C-terminal fusion protein, which had minimal PhoA activity, supports a GerUD topology where the TM-connecting loop is located on the external side of the membrane (Fig. S2, Supporting Information).

### GerUD structure–function relationships

Several mutant strains were designed and constructed in order to investigate structure–function relationships in the GerUD protein and to identify region(s) of the protein that are important to its function (Table S1, Supporting Information). In the first set of experiments, a series of *B. megaterium* strains were engineered to facilitate analysis of the importance of non-TM domain regions on GerUD function. Accordingly, strains were prepared bearing modified *gerUD* genes designed to result in proteins with (i) deletion of the predicted C- terminus residues [strain GC643] (ii) a C-terminus GFP fusion [strain GC644] (iii) a truncated (reduced to 3 residues) TM1–TM2 connective loop [strain GC645] and (iv) a GFP fusion in the TM1–TM2 connective loop [strain GC646]. Analysis of the kinetic parameters associated with spores of these strains, in terms of apparent affinity towards germinant (*K_m_*), maximal germinative rate (*V_max_*), and the concentration of germinants required to stimulate a 50% germinative response (*K_germ50_*), revealed that fusion of GFP to the loop or C-terminus had little effect on spore germination with respect to native GerUD-containing GC615 spores (Table 1). Unfortunately, neither of the variant GerUD-GFP proteins has yet proved useful in precisely localizing GerUD within the spore via western blot or fluorescence microscopy. Similarly, truncation of the loop that connects TM1 and TM2 to a minimal three-residue turn had little effect on the efficiency of germination in glucose or proline with respect to GC615 spores. Deletion of the short C-terminus cytoplasmic region was similarly largely inconsequential. Hence, from these experiments, we can conclude that neither the loop nor C-terminus cytoplasmic domain of GerUD is essential to its function, or to interactions with other components of the GerU receptor. As a caveat, the assumption throughout this work is that levels of variant GerUD proteins in spores are similar to those of the native GerUD protein in spores of the parental strain, although this may not be true. Indeed, neither the levels of GerUD in wild-type spores, nor the effect of varying those levels on GerU receptor function, have been established.

**Table 1.** Kinetic parameters of germination of *B. megaterium* GC614 (GR null) spores complemented with GerU containing variant GerUD proteins^a^.

		Kinetic parameter of germination in spores incubated with:					
		Glucose			Proline		
Strain	D-subunit protein	K*_m_*^b^	V*_max_*^c^	K*_germ50_*^^b^,^d^^	K*_m_*^b^	V*_max_*^c^	K*_germ50_*^^b^,^d^^
GC615	GerUD	1.7	0.05	0.24	1.7	0.05	0.24
GC631	None	7.5	0.04	2.5	5.4	0.04	2.5
GC643	GerUD with deleted C-terminal cytoplasmic region	1.6	0.06	0.24	1.6	0.06	0.24
GC645	GerUD with a truncated (3-residue) TM-spanning loop	1.3	0.05	0.22	1.3	0.06	0.22
GC644	GerUD–GFP	1.6	0.05	0.22	1.3	0.06	0.22
GC646	GerUD with GFP fused at the inter-TM-domain loop region	1.9	0.07	0.22	1.9	0.07	0.22

a Spores were germinated in 5 mM Tris-HCl, pH 7.5, for 90 min with concentrations of glucose and proline ranging from 0.1 to 1000 mM. Germination was monitored and kinetic parameters calculated as described in the methods section. Presented values for each strain are from three independent experiments conducted with the same spore preparation; similar values were obtained with at least one other spore preparation for each strain. Standard deviations from mean values are less than 10%.

b Values given in millimolar.

c Values represent the decrease in OD_600_ units/min, where starting values are normalized to 1 OD_600_ unit.

d Concentrations of glucose and proline required to stimulate 50% spore germination (*K_0.5germ_*) were calculated after incubation for 90 min with the respective germinants. Values presented for each strain are from three independent experiments conducted with the same spore preparation; similar values were obtained with other spore preparations. Standard deviations from mean values are less than 10%.

Next, the effect on germinative efficiency of substitution of *gerUD* within the *gerU* operon with genes encoding orthologous *B. megaterium* D-subunit proteins was examined. Previous work showed that substitution of *gerUD* with *gerKDbm* resulted in a marked decrease in the apparent function of the resultant modified GerU receptor (Ramirez-Peralta *et al*. 2013). Here, the effect of ectopic expression of various D-subunit genes, including *gerK_3_D* and BMQ_3896, which share high sequence identity at the amino acid level with GerUD (Fig. S3, Supporting Information) on the function of the GerU receptor was examined. The *gerK_3_D* gene is part of the operon that encodes the apparently non-functional GerK_3_ GR (Gupta *et al*. 2013), whereas BMQ_3896 is immediately adjacent to a gene predicted to encode a truncated GR B-subunit protein (BMQ_3895). Expression from both loci is expected to proceed as normal in the respective mutant backgrounds. Additionally, complementation with *B. subtilis gerKDbs*, which is more distantly related to *gerUD*, was examined also. Kinetic analysis of the resultant spores revealed that strain GC647, in which *gerUD* has been replaced with *gerKDbs* within the *gerU* operon, is associated with an ∼2-fold increase in apparent K*_m_* towards glucose or proline (Table 2). Since the maximal rate of germination of these spores is similar to the parental strain, these data indicate that the *gerKDbs* substitution is somehow exerting an influence on germinant binding. Conversely, spores of strain GC648, which have the closely related *gerK_3_D* in place of *gerUD*, showed a germination phenotype similar to that of the parental GC632 spores, indicating that the GerK_3_D protein can be tolerated within the GerU receptor without any adverse effects. However, spores engineered to express the D-subunit protein encoded at locus BMQ*_*3896 in place of GerUD (strain GC649) have a severe germination defect, despite the close similarity between the predicted proteins. Indeed, these spores failed to initiate germination in response to established single germinant compounds or a mixture of glucose, proline, leucine and KBr, but did retain a weak germinative response when incubated in 5% (w/v) beef extract (data not shown), indicating retention of a degree of GR functionality.

**Table 2.** Kinetic parameters of germination of *B. megaterium* GC614 (GR null) spores complemented with GerU containing various D-subunit orthologues^a^.

		Kinetic parameter of germination in spores incubated with:					
		Glucose			Proline		
Strain	D-subunit protein	K*_m_*^b^	V*_max_*^c^	K*_germ50_*^^b^,^d^^	K*_m_*^b^	V*_max_*^c^	K*_germ50_*^^b^,^d^^
GC615	GerUD	1.7	0.05	0.24	1.7	0.05	0.24
GC631	None	7.5	0.04	2.5	5.4	0.04	2.5
GC647	GerKDbs	2.8	0.06	0.37	2.3	0.06	0.34
GC648	GerK_3_D	1.3	0.07	0.21	1.2	0.06	0.21
GC649	BMQ_3896	NA^e^	NA	NA	NA	NA	NA
GC634	GerKDbm^f^	131	0.01	276	174	0.01	305

a Spores were germinated in 5 mM Tris-HCl, pH 7.5, for 90 min with concentrations of glucose and proline ranging from 0.1 to 1000 mM. Germination was monitored and kinetic parameters calculated as described in the methods section. Presented values for each strain are from three independent experiments conducted with the same spore preparation; similar values were obtained with at least one other spore preparation for each strain. Standard deviations from mean values are less than 10%.

b Values given in millimolar.

c Values represent the decrease in OD_600_ units/min, where starting values are normalized to 1 OD_600_ unit.

d Concentrations of glucose and proline required to stimulate 50% spore germination (*K_0.5germ_*) were calculated after incubation for 90 min with the respective germinants. Values presented for each strain are from three independent experiments conducted with the same spore preparation; similar values were obtained with other spore preparations. Standard deviations from mean values are less than 10%.

e NA, not applicable. These spores failed to germinate, precluding characterisation of kinetic parameters.

f Values from Ramirez-Peralta *et al*. (2013).

The structural and/or physiological basis of the deleterious effect of substitution of GerUD with BMQ_3896 is not clear. However, the presence of BMQ_3896, at least when expressed as part of the *gerU* operon, could feasibly interfere with the correct assembly of the GerU complex, or perhaps adversely affect the abundance of functional receptor proteins in the inner membrane. In order to investigate this possibility, a series of strains were constructed in which the GerUA protein was expressed with C- terminal 3X–FLAG or GFP epitopes, with a view to enabling receptor abundance via immunoblotting. Despite spores of various strains equipped with the GerUA fusion proteins germinating with efficiencies comparable to wild type spores—indicating that the proteins were expressed and the modified GerU receptors functional—polyclonal antisera against 3X–FLAG or GFP failed to detect the presence of the fusion proteins in purified spore inner membranes (data not shown). It seems likely that the western blot approach failed due to the extremely low abundance of the GerUA protein, and any future attempts to quantify receptor abundance will probably require antisera against GerUA as opposed to a relatively small fusion-derived epitope.

In the final series of experiments, the importance of individual putative TM domains to the function of GerUD was examined. This was achieved by designing a series of fusion constructs in which either TM1 or TM2 of GerUD was replaced with the corresponding TM domain from GerKDbm, GerKDbs or BMQ_3896 (Table S1, Supporting Information). Additionally, strains were engineered in which the predicted loop regions from the same orthologous D-subunit proteins were introduced to replace the corresponding loop in GerUD. Finally, strains engineered to express only single TM domains (TM1 or TM2) plus short loop sections from the various orthologues were designed and prepared also. Sporulation of all strains was observed to be normal, following which the germinative efficiency of the various mutants was monitored in response to glucose and proline.

Unexpectedly, all mutant constructs bearing modified GerUD proteins displayed germination phenotypes and kinetic values that were broadly in line with the native GerUD-containing GC632 strain (Table 3). This includes fusion proteins that contain either TM1 or TM2 from BMQ_3896, which when present as the full-length protein in the ΔGerUD background causes a severe germination defect. Hence, it seems that the presence of either GerUD TM1 or TM2 can compensate for the deleterious effect of the individual BMQ_3896 TM domains. Additionally, these data reveal that GerUD can tolerate relatively major substitutions to individual TM (and loop) domains without adversely affecting its function.

**Table 3.** Kinetic parameters of germination of *B. megaterium* GC614 (GR null) spores complemented with GerU containing truncated, fusion and variant D-subunit proteins^a^.

		Kinetic parameter of germination in spores incubated with:					
		Glucose			Proline		
Strain	D-subunit protein	K*_m_*^b^	V*_max_*^c^	K*_germ50_*^^b^,^d^^	K*_m_*^b^	V*_max_*^c^	K*_germ50_*^^b^,^d^^
GC615	GerUD	1.7	0.05	0.24	1.7	0.05	0.24
GC631	None	7.5	0.04	2.5	5.4	0.04	2.5
GC665	GerKDbm TM1 GerUD TM2	2.0	0.05	0.28	1.9	0.05	0.27
GC666	GerUD TM1 and TM2 connected by GerKDbm loop	2.0	0.05	0.27	1.9	0.05	0.26
GC667	GerUD TM1 GerKDbm TM2	1.8	0.05	0.25	1.4	0.05	0.22
GC671	BMQ_3896 TM1 GerUD TM2	2.0	0.05	0.25	1.9	0.06	0.24
GC672	GerUD with BMQ_3896 loop	1.7	0.06	0.24	2.0	0.07	0.25
GC673	GerUD TM1 BMQ_3896 TM2	1.7	0.05	0.23	1.8	0.05	0.24
GC668	GerKDbs TM1 GerUD TM2	1.5	0.05	0.22	1.7	0.05	0.24
GC669	GerUD with GerKDbs loop	1.8	0.05	0.24	1.7	0.05	0.24
GC670	GerUD TM1 GerKDbs TM2	1.9	0.05	0.24	1.8	0.05	0.24
GC638	GerUD (amino acid sequence reversed)	1.9	0.06	0.25	1.7	0.07	0.24
GC639	N-GerUD TM2 GerUD TM1-C	2.0	0.06	0.24	1.6	0.06	0.23
GC674	GerUD TM1	1.8	0.08	0.22	2.2	0.07	0.27
GC675	GerUD TM2	1.9	0.08	0.24	1.9	0.08	0.25
GC676	BMQ_3896 TM1	2.0	0.06	0.26	2.1	0.07	0.25
GC677	BMQ_3896 TM2	1.9	0.06	0.24	2.0	0.07	0.24

a Spores were germinated in 5 mM Tris-HCl, pH 7.5, for 90 min with concentrations of glucose and proline ranging from 0.1 to 1000 mM. Germination was monitored and kinetic parameters calculated as described in the methods section. Presented values for each strain are from three independent experiments conducted with the same spore preparation; similar values were obtained with at least one other spore preparation for each strain. Standard deviations from mean values are less than 10%.

b Values given in millimolar.

c Values represent the decrease in OD_600_ units/min, where starting values are normalized to 1 OD_600_ unit.

d Concentrations of glucose and proline required to stimulate 50% spore germination (*K_0.5germ_*) were calculated after incubation for 90 min with the respective germinants. Values presented for each strain are from three independent experiments conducted with the same spore preparation; similar values were obtained with other spore preparations. Standard deviations from mean values are less than 10%.

Similarly, spores complemented with GerUD variants in which the order of the TM domains was reversed (TM2 at the N-terminal and TM1 at the C-terminus; strain GC639), for example, were observed to germinate relatively efficiently. More surprisingly, spores complemented with a GerUD variant in which the entire amino acid sequence was reversed (GC638) also germinated relatively normally. Both of these reverse/inverse D-subunit proteins are expected to comprise a two-TM domain structure, but presumably with different topology and or orientation, indicating that the mere presence of the helices is commensurate with the proteins’ function. The hypothesis that a single TM helix is sufficient to confer D-subunit function, regardless of orientation of the helix in the membrane, was strengthened further by experiments with spores complemented with a series of truncated (single TM) D-proteins. Hence, strains GC674 and GC675, which contain single GerUD TM helices, and GC676 and GC677, which contain single BMQ_3896 TM helices, germinate relatively normally with respect to GC632 spores (Table 3). From these data, we can infer that the deleterious effect of BMQ_3896 results from a presumed structural incompatibility when both TM domains are present. It seems likely that this is at the protein interaction level, although whether these are adverse homo– (i.e. D-subunit) or hetero-(D-subunit–GR protein) interactions remains to be elucidated.

To conclude, results from the current study are consistent with the GerUD protein, and by inference other GR D-subunit proteins, being a spore inner-membrane located protein comprising two TM domains that are connected by an integument-facing loop, and spore-core located N- and C- termini. Kinetic analysis of mutant strains bearing variant GerUD proteins have revealed that the GerUD protein can be subjected to severe truncation and or substitution while apparently retaining function. Indeed, the presence of a single TM domain–either TM1 or TM2–is adequate for maintenance of an efficient germinative response mediated via the GerU receptor. However, the precise function of D-subunit proteins remains unknown, although one possibility is that they act as molecular chaperones, facilitating folding or minimizing aggregation of GR proteins within the spore inner-membrane environment. A key objective of future work in this area will be to establish whether D-subunit proteins interact, perhaps in the suggested chaperone-type capacity, with other subunits of associated GRs, or with accessory proteins, such as GerD.

## Supplementary Material

Supplementary data are available at FEMSLE online
