# Supplementary material for: Structure–function analysis of the Bacillus megaterium GerUD spore germinant receptor protein
Source: FEMS Microbiol Lett. 2015 Oct 28;362(24):fnv210. doi: 10.1093/femsle/fnv210 (PMC4661057; doi:10.1093/femsle/fnv210)
Supplement: Supplementary data are available at FEMSLE online [file Supplemental_Information.docx]

**Supplemental Information**

**Table S1** *Bacillus megaterium* strains employed in this work

| Strain*^1^* | Relevant phenotype and genotype*^2^* | Source |
| --- | --- | --- |
| GC614 | Ger_null_ (Δ*gerU* Δ*gerK* Δ*gerK_2_* Δ*gerA* Δ*gerA_2_*) Km^r^ MLS^r^ Cm^r^ Sp^r^ | Gupta *et al*., 2013 |
| GC614 derived strains*^3^* | | |
| GC615 | Ger_null_ pHT-*gerUD* *gerU***^4^* | Ramirez-Peralta *et al*. 2013 |
| GC631 | Ger_null_ pHT-*gerUD*^M10stop^ *gerU** | Ramirez-Peralta *et al*. 2013 |
| GC632 | Ger_null_ pHT-*gerUD*^M10stop^ *gerU**, with *gerUD* located between *gerUC* and *gerVB* | Ramirez-Peralta *et al*. 2013 |
| GC643 | Ger_null_ pHT-*gerUD*^M10stop^ *gerU**, with *gerUD* (codons 1 - 68 i.e. C-terminal deletion) between *gerUC* and *gerVB* | This work |
| GC644 | Ger_null_ pHT-*gerUD*^M10stop^ *gerU**, with *gerUD-gfp* (GFP fused after codon 70) between *gerUC* and *gerVB* | This work |
| GC645 | Ger_null_ pHT-*gerUD*^M10stop^ *gerU**, with *gerUD* (encoding GerUD with a truncated loop [codons 26-43 deleted]) between *gerUC* and *gerVB* | This work |
| GC646 | Ger_null_ pHT-*gerUD*^M10stop^ *gerU**, with *gerUD-gfp* (where *gfp* is located in the predicted loop [between codons 26 - 43]) between *gerUC* and *gerVB* | This work |
| GC647 | Ger_null_ pHT- *gerUD*^M10stop^ *gerU**, with *gerKDbs* located between *gerUC* and *gerVB* | This work |
| GC648 | Ger_null_ pHT-*gerUD*^M10stop^ *gerU**, with *gerK_3_D* located between *gerUC* and *gerVB* | This work |
| GC649 | Ger_null_ pHT-*gerUD*^M10stop^ *gerU**, with *BMQ_3896* located between *gerUC* and *gerVB* | This work |
| GC634 | Ger_null_ pHT- *gerUD*^M10stop^ *gerU**, with *gerKDbm* located between *gerUC* and *gerVB* | Ramirez-Peralta *et al*. 2013 |
| GC665 | Ger_null_ pHT-*gerUD^M10stop^ gerU**, *gerUD* (with GerKDbm TM1 [codons 4-22] instead of GerUD TM1 [codons 6-24]) between *gerUC* and *gerVB* | This work |
| GC666 | Ger_null_ pHT-*gerUD^M10stop^ gerU**, *gerUD* (with GerKDbm loop [codons 23-30] instead of GerUD loop [codons 26-43]) between *gerUC* and *gerVB* | This work |
| GC667 | Ger_null_ pHT-*gerUD^M10stop^ gerU**, *gerUD* (with GerKDbm TM2 [codons 31-50] instead of GerUD TM2 [codons 45 – 67]) between *gerUC* and *gerVB* | This work |
| GC668 | Ger_null_ pHT-*gerUD^M10stop^ gerU**, *gerUD* (with GerKDbs TM1 [codons 3-24] instead of GerUD TM1 [codons 5-25]) between *gerUC* and *gerVB* | This work |
| GC669 | Ger_null_ pHT-*gerUD^M10stop^ gerU**, *gerUD* (with GerKDbs loop [codons 25-44] instead of GerUD loop [codons 26–43]) between *gerUC* and *gerVB* | This work |
| GC670 | Ger_null_ pHT-*gerUD^M10stop^ gerU**, *gerUD* (with GerKDbs TM2 [codons 45-66] instead of GerUD TM2 [codons 45-67]) between *gerUC* and *gerVB* | This work |
| GC671 | Ger_null_ pHT-*gerUD^M10stop^ gerU**, *gerUD* (with BMQ_3896 TM1 [codons 6-24] instead of GerUD TM1 [codons 5-25]) between *gerUC* and *gerVB* | This work |
| GC672 | Ger_null_ pHT-*gerUD^M10stop^ gerU**, *gerUD* (with BMQ_3896 loop [codons 25-44] instead of GerUD loop [codons 26-43]) between *gerUC* and *gerVB* | This work |
| GC673 | Ger_null_ pHT-*gerUD^M10stop^ gerU**, *gerUD* (with BMQ_3896 TM2 [codons 45-67] instead of GerUD TM2 [codons 45-67]) between *gerUC* and *gerVB* | This work |
| GC674 | Ger_null_ pHT-*gerUD^M10stop^ gerU**, *gerUD* (codons 1-34 i.e. N-terminal and TM1 only) between *gerUC* and *gerVB* | This work |
| GC675 | Ger_null_ pHT-*gerUD^M10stop^ gerU**, *gerUD* (encoding N-terminal [codons 1-5] and TM2 [codons 45-67] only) between *gerUC* and *gerVB* | This work |
| GC676 | Ger_null_ pHT-*gerUD^M10stop^ gerU**, *BMQ_3896* (codons 1-30 [N-terminal and TM1) between *gerUC* and *gerVB* | This work |
| GC677 | Ger_null_ pHT-*gerUD^M10stop^ gerU**, *BMQ_3896* (codons 1-5 [N-terminal] fused to codons 45-67 [TM2]) between *gerUC* and *gerVB* | This work |
| GC639 | Ger_null_ pHT-*gerUD*^M10stop^ *gerU**, *gerUD* (with N-terminal [1-5]-TM2 [45-67] -loop [25-44] -TM1 [6-24] -C-terminal [68-76]) between *gerUC* and *gerVB* | This work |
| GC638 | Ger_null_ pHT-*gerUD*^M10stop^ *gerU**, *gerUD* (where residues 2-76 are encoded in the reverse order) between *gerUC* and *gerVB* | This work |

^1^ All strains are isogenic with *B. megaterium* PV361, a plasmidless derivative of the QM B1551 wild type strain.

^2^Abbreviations for antibiotics are: Km^r^, kanamycin resistance (5 µg/ml); Sp^r^, spectinomycin resistance (100 µg/ml); Cm^r^, chloramphenicol resistance (5 µg/ml); MLS^r^, resistance to erythromycin (1 µg/ml) plus lincomycin (25 µg/ml); Tc^r^, tetracycline resistance (12.5 µg/ml).

^3^ These strains have the GC614 background, and were transformed to Tc^r^ with plasmid pHT315t carrying the described receptor genes. All strains are Km^r^ MLS^r^ Cm^r^ Sp^r^ Tc^r^

^4^ *gerU**, GR operon comprising *gerUA*, *gerUC* and *gerVB*

**Table S2 Transmembrane domain predictions for various D-subunit proteins*^1^***

| **D–protein** | **TM1***^2^* | | **TM2***^2^* | |
| --- | --- | --- | --- | --- |
|  | Start | End | Start | End |
| GerUD | 6 | 24 | 45 | 67 |
| GerKDbm | 4 | 22 | 31 | 50 |
| GerK_3_D | 7 | 25 | 46 | 68 |
| BMQ_3896 | 6 | 24 | 45 | 67 |
| GerKDbs | 3 | 24 | 45 | 66 |

*^1^* The following web-based programs were used to arrive at predicted amino acid positions for TM-spanning domains: SOSUI, PHD Predict Protein, Split 4.0, TMHMM, TMPred, TopPred, TMMOD, MEMSAT-SVM, MEMSAT3, HMMTOP, TOPCONS.

*^2^* Start and End refers to consensus amino-acid positions predicted to encode D-subunit TM-spanning domains.

**Figure S1**

**
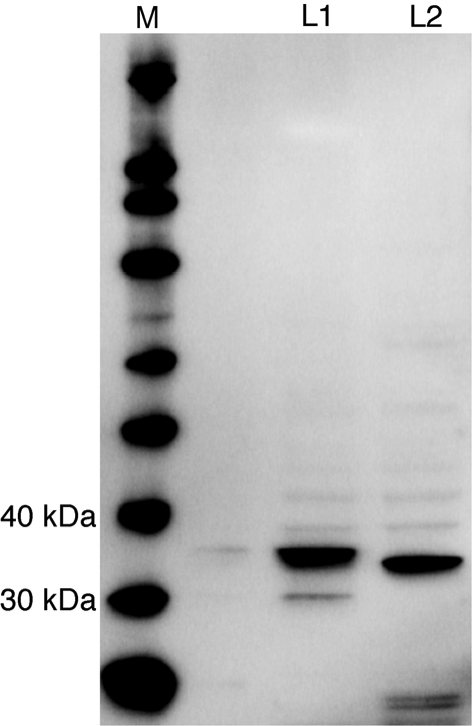
**

**Figure S1** Western blot of GerUD-GFP proteins. Equivalent samples of membrane fractions from *E. coli* cells expressing GerUD-GFP proteins were subject to SDS-PAGE, transferred to a PVDF membrane and then blotted with anti-GFP antisera. Key: M, molecular mass marker; L1, GerUD with a C-terminus GFP fusion; L2, GerUD with GFP fused in the predicted loop region (between residues 26 and 43).

**Figure S2**


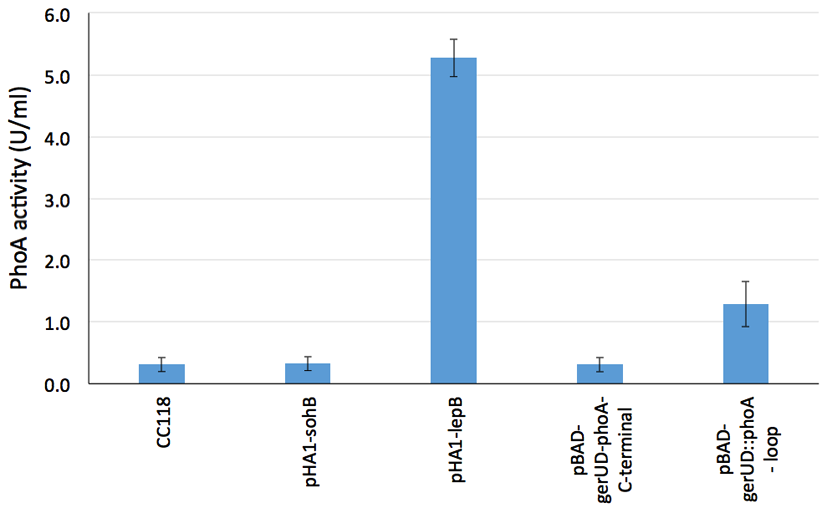


**Figure S2** Alkaline phosphatase activity associated with *E. coli* CC118 (Δ*phoA* Δ[*ara-leu*]) expressing PhoA fusion proteins. Activity was assayed by measuring the ability of intact cells to hydrolyse *p*-nitrophenyl phosphate to *p*-nitrophenol and inorganic phosphate, with the associated change in absorbance at 405 nm measured over a 15-minute incubation period. Cells containing plasmids pHA1-sohB and pHA1-lepB were used as negative and positive controls respectively (courtesy of Dan Daley, Stockholm University). Enhanced PhoA activity in the GerUD-PhoA loop construct compared to the C-terminal fusion construct, which is statistically significant (*P*<0.005, as determined by two-tailed Student’s *t* test), is indicative of a periplasmic location for the loop region of the protein. Data represent the means ± standard deviations of three independent experiments.

**Figure S3**


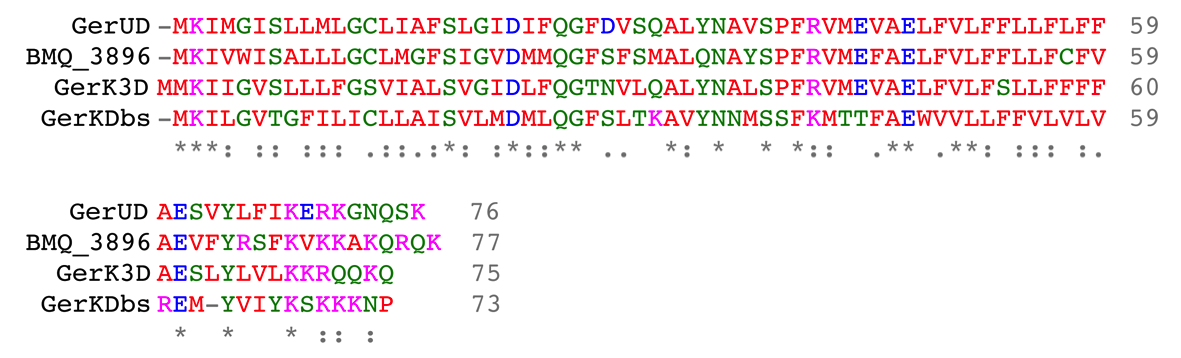


**Figure S3** Clustal Omega alignment of *B. megaterium* GerUD, BMQ_3896, and GerK_3_D, and *B. subtilis* GerKD (GerKDbs). The fifth D-subunit protein used in this work, *B. megaterium* GerKD, shares 14-20 % sequence identity at the amino acid level with the other D-subunit proteins and shows no discernible sequence alignment by this method.
